# Personality Traits in Patients with Subjective Idiopathic Tinnitus

**Published:** 2015-09

**Authors:** Mahboobeh Adami Dehkordi, Maryam Javanbakht, Shima Sarfarazi Moghadam, Mojtaba Meshkat, Samaneh Abolbashari

**Affiliations:** 1 *Department of Otorhinolaryngology, Mashhad Medical Sciences Branch, Islamic Azad University, Mashhad, Iran.*; 2 *Department of Psychology, Mashhad Medical Sciences Branch, Islamic Azad University, Mashhad, Iran.*; 3 *General Practitioner, Mashhad Medical Sciences Branch, Islamic Azad University, Mashhad, Iran. *; 4 *Department of Biostatistics, Mashhad Medical Sciences Branch, Islamic Azad University, Mashhad, Iran.*

**Keywords:** Psychological characteristics, Personality traits, Tinnitus

## Abstract

**Introduction:**

Tinnitus is a common complaint in patients referred to otorhinolaryngology clinics and is a condition where one hears a sound without any distinguishable external acoustic source or electrical stimulus. About 3-30% of adults experience different degrees of tinnitus during their life. This study aims to ascertain and compare personality traits between patients with tinnitus and a control group.

**Materials and Methods:**

In a case control study, 66 participants were assessed. The case group consisted of 33 patients who suffered from tinnitus for at least two months, in addition to 33 healthy volunteers who were selected among their family (preferably of the same age and sex). A standard demographic questionnaire and an Eyzenck personality questionnaire were filled for both groups. A tinnitus severity index (TSI) questionnaire was only filled for the case group. Data from each group was compared by Mann-Whitney U and Chi-Square tests. SPSS V.18 was the selected software.

**Results:**

Statistical analysis showed a meaningful difference in neuroticism (P=0.001) and extraversion (P=0.001) between the patients and the controls; however, there was no statistical difference between these groups regarding psychotism.

**Conclusion:**

Tinnitus can be associated with personality characteristics. This study showed that in patients with tinnitus, neuroticism increases and extraversion decreases. Considering the personality and psychotic traits observed in the patients with tinnitus, psychiatric consultation is recommended.

## Introduction

The term tinnitus refers to the perception of a sound in the ears or head without external stimulus (1-3). Although the prevalence of tinnitus is not uncommon, the severity of the audible sound varies from unnoticeable to very severe. About 35-40 percent of adults in industrial countries experience tinnitus one time in their life (4). 

Tinnitus has a wide spectrum of effects on patients (5). In most adults, it is not intolerable. In only 3-5 percent of patients, tinnitus is annoying enough to produce distractive effects on daily activities, general health, and sleep (1).

Personality and coping mechanisms affect one's perception of tinnitus. There is a relation between stress and tinnitus that cause some of the patients to experience severe stress (6). Loud and continuous tinnitus can be highly worrisome and can produce psychological problems and even sometimes lead to suicide in some patients (7). 

On the other hand, neurotic tensions can increase tinnitus. In this case; its severity would be higher at the end of day. Tinnitus has several consequences such as insomnia, problems in attention and concentration, increased sensitivity to noise, and negativism. Based on previous studies, there is a direct relation between tinnitus and occurrence of psychological disorders, which also indirectly affect the patient’s family. Some patients may show severe degrees of depression or be defensive about their tinnitus when brought up in conversation.

Most patients benefit from psychiatric interventions (7). The connection between tinnitus and personality types has been evaluated in previous studies (5).

However, the relation between tinnitus and personality traits has not been reported in any other study; therefore this study was conducted to evaluate personality traits in our patients. 

## Materials and Methods

*Study design:* In a case-control study we assessed 33 patients with idiopathic tinnitus as a case group and 33 health volunteers as a control group. Patients with tinnitus, who were older than 18 years old and had been referred to the otorhinolaryngology Department of 22^nd ^Bahman Hospital in Mashhad-Iran during 2010-2012, were chosen as the case group. All patients suffered from non-pulsatile, subjective, and idiopathic tinnitus for at least 2 months without any kind of hearing loss or vertigo. Based on history, physical exam, audiology tests, and MRI, patients with a known etiology for tinnitus such as meniere, chronic otitis media, acustic neurinoma, etc were excluded. Thirty-three healthy volunteers from the patients’ family, who matched them in age and sex, were included as a control group.

***Clinical Procedure***

During the first visit, a complete history was taken from each patient. After, an otology examination was performed. Audiology tests including pure tone audiometry (PTA), speech reception threshold (SRT), speech discrimination score (SDS), and tinnitus evaluation test (TET), were administered for all patients with inclusive criteria (age older than 18 years, having non-pulsatile tinnitus for at least 2 months, absence of tympanic membrane perforation). To rule out any distinguishable lesions such as acoustic neurinoma and multiple sclerosis, brain MRI (Magnetic resonance imaging) without and with contrast, was administered for all participants in the case group. Patients who met all the inclusive criteria filled a questionnaire which consisted of demographic information, the duration of tinnitus, the type of sound, accompanying symptoms, history of systemic disease, and disability caused by tinnitus. Tinnitus severity index (TSI) was used to measure the impact of tinnitus on the patient's quality of life. TSI consists of 12 questions, questions 1-9 with 5, 10 with 3, and 11-12 with 4 possible answers and a total score, which ranges from 12 to 56. The loudness of tinnitus was assessed with a one to ten scale and patients were asked to choose a number that was best compatible with the loudness of the sound they heard. The scores were divided into three groups: mild, moderate, and severe (1-4, 5-6 and 7-10 respectively).

Finally, to evaluate the personality traits, participants in both groups filled the Eyseneck questionnaire. This questionnaire has 90 questions consisting of 21, 25, 23, and 21 questions to assess the traits of extraversion, neuroticism, and psychotism respectively. Each question has two possible answers (yes or no). Based on test directory for scoring, in some questions the answer for YES is gain 1 and NO is gain 0; however, in some other questions it is the opposite. The validity and reliability of the Persian version of Eysneck questionnaire has been evaluated in Iranian population and satisfactory results were obtained. (8)

The normality of the data was evaluated with Kolmogorov-Smirnov test. The comparison of the two groups was achieved through the Mann-Whitney U and Chi-Square tests. The correlation of the data was assessed with Spearman’s correlation index. SPSS-v.18 was the selected software and the meaningful level of for the test is considered to be less than 0.05.

## Results

In this study 33 patients with subjective idiopathic tinnitus (case group) and 33 healthy volunteers selected from their family (control group) were assessed. The mean age in the case and control groups was 52.9±13.4 and 56.2±9.7 respectively. The two groups were not statistically different in age, sex, marriage and education (Table. 1).

**Table1:** Characteristics of participants

Variable	Case (n=33)	Control (n=33)	P-Value
**Age (year)**	52.9±13.4	56.2±9.7	0.220^a^
**Sex (male\female)**	15\18	15\18	1.000^b^
**Marriage ** **(single\married)**	6\27	4\29	0.733 b
**Education (academic\nonacademic)**	9/24	11/22	0.592 b


a ) Mann-Whitney U test,

b )chi-square test

The case group consisted of 18 females and 15 males. Based on a 1-10 score, 3 (9.1%) patients scaled their tinnitus as mild, 10 (30.3%) as moderate, and 20 (60.6%) as severe. Although there was no significant difference between the two sexes in the case group (p=0.06), the severity of tinnitus was higher in women. In this group, 77.8% of women had severe tinnitus (7-10), whereas the most common category of tinnitus intensity for men (46.7%) was medium(4-6). This difference was not statistically significant (P=0.067). Evaluation of correlation between subjective assessment of tinnitus, based on a 1-10 scale score and the tinnitus severity index (TSI), showed a linear direct relation which was statistically significant (P=0.003 - r=0.51). TSI in patients divided into 5 groups consists of 20-25, 26- 31, 32-37, 38 – 43, 44 and more. The annoyance of tinnitus based on TSI had no significant difference in men and women (P=0.155).

Although personality traits analysis showed no significant difference between the two groups with regard to psychoticism (P=0.295); neuroticism and extraversion showed a significant difference between the two groups (P=0.001 and P=0.001 respectively). This means that the mean score of neuroticism in the case group was 16.4±4.2, which is significantly higher than the mean score in the control group. (10.9±4.1). The mean score of extraversion in patients was 9.6-+3.6, which is significantly lower than that of the control group (14.2 ± 2.8) (Table. 2).

**Table2:** Comparison of Neuroticism, Psychotism, and Extraversion in each group

Personality traits	Case (n=33)	Control (n=33)	P-Value
**Neuroticism**	16.4±4.2	10.9±4.1	0.001^a^
**Psychotism**	4.3±2.2	3.6±1.4	0.295^a^
**Extraversion**	9.6±3.6	14.6±2.8	0.001^a^


a ) Mann-Whitney U test

The relation between personality traits in patients and age, sex, marriage, TSI (on a 1-10 scale score), duration of tinnitus, sleeping problem, problems in attention and concentration, and problems in daily activity are given in (Table.3). There was a significant difference between the three personality traits regarding the duration of tinnitus, sleeping problems, concentration problems, and problems in daily activity. 

**Table 3:** The main demographic and clinical characteristics in each personality group

Personality traits	Neuroticism Mean±SD P-Value		Psychotism Mean±SD P-Value		Extraversion Mean±SD P-Value	
**Age** **30-** ** 30-39** ** 40-49** ** 50-59** ** 60 +**	13.2±3.0 13.7±3.0 12.7±4.3 11.2±4.4 9.4±4.0	0.147 b	13.2±4.5 10.7±5.6 15.2±4.0 13.2±4.5 15.5±6.1	0.132 b	3.9±1.1 3.4±.7 4.3±1.7 3.5±2.3 4.7±2.5	0.303 b
**Sex ** **-) Male** ** -) Female**	13.1±3.8 11.2±4.2	0.056 a	11.9±4.7 15.3±4.7	0.004 a	3.7±1.8 4.1±1.9	0.397 a
**Marriage ** **-) single** **-) married**	12.9±3.0 11.9±4.3	0.653 a	14.7±5.7 13.5±4.8	0.472 a	4.1±1.2 3.9±2.0	0.587 a
**Education ** ** -) academic** ** -) nonacademic**	11.7±4.1 13.0±4.0	0.280 a	14.3±5.2 12.3±4.1	0.057 a	4.2±1.9 3.5±1.7	0.179 a
**Duration ** ** 4-** ** 4-6** ** 6+**	14.6±2.8 10.2±4.3 8.8±2.5	0.0001 b	11.0±4.1 15.8±5.1 17.4±2.4	0.0001 b	3.6±1.4 4.1±2.0 4.6±2.4	0.569 b
**Sleeping problem** ** -) No** ** -) Yes**	13.5±3.8 9.5±3.3	0.0001 a	12.0±4.8 16.9±3.4	0.0001^ a^	3.5±1.7 4.7±1.9	0.010 a
**Concentration problem** ** -) No** ** -)Yes**	13.2±3.9 9.5±3.3	0.001 a	12.4±4.7 16.8±4.3	0.001 a	3.7±1.6 4.5±2.3	0.224 a
**Daily activity problem ** ** -)No** ** -)Yes**	12.6±4.2 9.6±2.6	0.024 a	13.1±4.8 16.9±4.6	0.016 a	3.7±1.6 5.3±2.3	0.035 a

a )Mann-Whitney U test

b )Kruskal Wallis Test

## Discussion

During this case control study we tried to evaluate personality traits in patients with subjective idiopathic tinnitus. Our study showed higher levels of neuroticism and lower levels of extraversion in patients compared to controls.

Neuroticism is a personality trait that is characterized by anxiety, fear, worry, moodiness, and envy. Individuals with a high score in neuroticism experience more anxiety, envy, anger, guilt, and depression, in addition to having an ineffective coping mechanism for stress. 

Extraversion tends to be found in outgoing, talkative, assertive, energetic, and gregarious people, whereas introversion tends to be found in more reserved and solitary people.

Individuals with neuroticism and introversion tend to experience negative emotions like sadness and experience a dark life, which can be associated with tinnitus. 

The same conclusion was reported by Bartels et al (5). In this present study, the case group consisted of 18 women and 15 men. Although the number of women is higher than men, it isn't statistically significant (P=o.6). The same conclusion was reported by Welch et al (4). They interviewed a birth cohort of 670 people, which were aged at 32 years and were sampled from the general population. The population was divided into three groups, those without tinnitus, those with occasional tinnitus, and those who experienced tinnitus most of the time. They concluded that men and women didn't differ in the amount of tinnitus they suffered from, but women were more likely to find it annoying. On the other hand, during a case-control study on 265 patients with tinnitus (case group) and 265 Ear Nose and Throat (ENT) patients without tinnitus (control group), Bartel et al reported that the tinnitus group comprised of more men compared to the control group (5). In the present study we matched the two groups regarding sex and age. The control group didn't have any knowable ENT disease. Of course, in Bartel’s study, the number of men in the tinnitus group was higher than the number of women, which was statistically significant. Our case number is lower than the Bartel study’s case number, which can explain this difference. 

Although Welch et al concluded that the severity of annoyance secondary to tinnitus is higher in women than in men (4), we didn't observe any significant difference between men and women from this point of view (P=0.155), This could be because of the way annoyance is assessed. We used the TSI questionnaire and annoyance was assessed by asking the question "How annoying or upsetting is it?” with four possible answers: "not at all", "slightly", "moderately" and "severely". 

We evaluated the relation between TSI and the 1-10 tinnitus scale score also in addition to the relation of each with each personality trait.

There was a statistically significant relation between TSI and the 1-10 scale score (P=0.003). This item is not assessed in any similar study. It means that the patients’ self-assessment of tinnitus is compatible with the result of TSI.

Although the results of Bartle et al’s study showed that personality traits, especially type D personality (5), may have a relation with tinnitus and might contribute to its perceived severity, we didn't find any relation between TSI or the 1-10 tinnitus scale score with any personality traits. The method for evaluating tinnitus severity in Bartel’s study is not clear. In the present study the severity of tinnitus was assessed with TET in addition to the 1-10 scale score and TSI. In addition, since Type D patients are susceptible to experiencing a wide range of negative emotions, including anxiety and depression, when confronted with overpowering health problems such as tinnitus, patients with this personality taxonomy may experience more severe tinnitus (5).

The mean duration of disease in our patients was 4.5 years, which had no significant relation with each personality traits. Krog NH et al claimed that patients with chronic tinnitus experienced higher degrees of depression and anxiety (10). They observed diminished self-confidence and a feeling of well being in their patients. Since personality traits are almost fixed during life, it is predictable that the duration of disease can't cause any difference in personality. Anxiety and depression are types of psychiatric disorders that can occur after a chronic somatogenic disease.

We used the Eysenbeck questionnaire to evaluate personality traits. This questionnaire has 90 questions consisting of 21,25,23, and 21 questions to assess each trait of extraversion, neuroticism, and psychotism. In accordance with Welch et al (4), the results of the given study demonstrate that neuroticism has a higher frequency in all classes of age, sex, education, and marriage in the tinnitus patient group while psychotism has a lower frequency. Welch thought that different personality traits affect the bias to report tinnitus. Although the approaches of these two studies are different, the results are similar. Welch et al carried out a cross-sectional study on a young population in order to observe the effect of personality on a future experience of tinnitus.

18 (50.4%) of our patients suffered from sleeping problems caused by tinnitus. The severity of tinnitus was severe in 10 patients and moderate in 8 patients. The obtained results confirmed a relation between sleeping problems and the severity of tinnitus (P=0.04) and Neuroticism (P=0.014). Therefore the severity of tinnitus is an important factor in inducing sleeping problems. In addition, Langenbech M et al concluded that initial insomnia is one of the factors (6), which has the highest predictive value for the development of tinnitus distress. It means that sleeping problems can be produced by tinnitus and that tinnitus itself may be a factor leading to its development.

Concentration problems were observed in 20 (66.6%) of our patients (12 with severe, 7 with moderate, and 1 with mild tinnitus). There was no relation between concentration problems and tinnitus severity or personality traits.

This item was not assessed in any similar study. 

Although eleven patients (33.3%) experienced daily activity problems, there was no significant relation with the severity of tinnitus and personality traits. In Valianatou’s study - similar to the present survey- most of the patients adapted to their tinnitus and had no problem in their daily activities. On the other hand, unlike this study, Marciano et al found that tinnitus had a significant effect on the patient’s functions in some aspects of their life (11). This difference may be caused by fewer cases in the present research or the difference in the study design. In Marciano et al’s study, patients had some additional symptoms like sensory neural hearing loss and vertigo, which can diminish daily activity. However, in the present study, our patients only had tinnitus without any concomitant symptoms. 

## Conclusion

This study found that tinnitus patients had a tendency to be more neurotic and less extraverted than others. This suggests that they may benefit from psychiatric consultations. It is important to consider the psychopathologic relations in patients with tinnitus in the process of their treatment and follow up.
